# P-529. Comparison of Renal Outcomes by Tenofovir Alafenamide Fumarate (TAF) vs. Tenofovir Disoproxil Fumarate (TDF) Containing Regimens for Prevention, and Treatment of HIV and/or HBV Treatment: A Systematic Literature Review and Meta-Analysis

**DOI:** 10.1093/ofid/ofae631.728

**Published:** 2025-01-29

**Authors:** Xi Liang, Kyu Yun Park, Haeseon Lee, Connor Willis, Tara Dhippayom, Rachel Rogers, Amy Weinberg, Julia Green, Aileen Chi, Nathorn Chaiyakunapruk

**Affiliations:** University of Utah College of Pharmacy, Salt Lake City, Utah; University of Utah College of Pharmacy, Salt Lake City, Utah; University of Utah College of Pharmacy, Salt Lake City, Utah; University of Utah College of Pharmacy, Salt Lake City, Utah; The Prince Royal's College, Chiang Mai, Chiang Mai, Thailand; Gilead Sciences, Inc., Foster City, California; Gilead Sciences, Inc., Foster City, California; Gilead Sciences, Inc., Foster City, California; University of Utah, Salt Lake City, Utah

## Abstract

**Background:**

Tenofovir is one of the most widely used agents for human immunodeficiency virus (HIV) and hepatitis B virus (HBV) treatment, and pre-exposure prophylaxis (PrEP). Two forms of tenofovir are currently available: tenofovir disoproxil fumarate (TDF) and its prodrug, tenofovir alafenamide (TAF). TAF is associated with improved renal safety and non-inferior efficacy when compared to TDF. We aimed to comprehensively synthesize the evidence on renal outcomes of TDF and TAF regimens for treatment of HIV-1, HBV, or PrEP.

**Figure:**
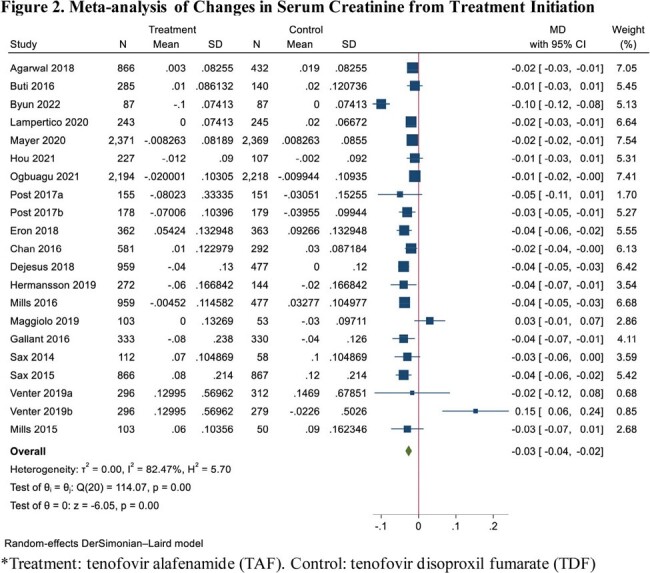

**Methods:**

A systematic search of randomized controlled trials (RCTs) that compared TDF vs. TAF for the treatment of HIV-1, HBV, or PrEP and reported changes in estimated glomerular filtration rate (eGFR) or serum creatinine (SCr) from treatment initiation was conducted in PubMed, EMBASE, Web of Science, and the Cochrane Trial Registry. This search was performed on 12/6/2023 and supplemented a previous review by Gupta et al. (2019). Renal outcomes were pooled using mean differences with 95% confidence intervals (CIs). The heterogeneity was assessed using I^2^ test and explored via subgroup analyses of disease state and duration of follow-up.

**Figure:**
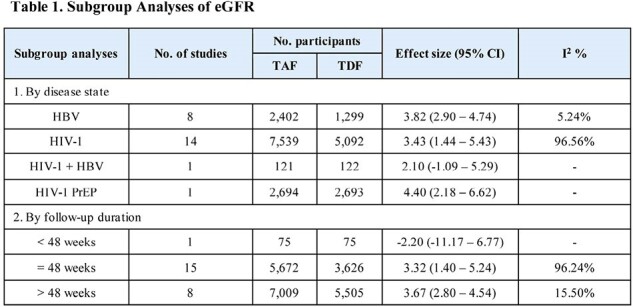

**Results:**

Out of 9,014 studies screened, 24 RCTs (total 21962 participants; 12756 in TAF; 9206 in TDF) reported eGFR outcomes and 21 RCTs (total 18454 participants; 10293 in TAF; 8161 in TDF) reported SCr outcomes. Compared to TDF, the use of TAF was associated with an improvement of 3.69 mL/min/1.73m^2^ (95% CI: 1.89 – 5.49) in eGFR from treatment initiation (I^2^ = 95.83%) (Figure 1). Subgroup analyses of disease state and study duration for eGFR are described in Table 1. The effect sizes were consistent across subgroups, low levels of heterogeneity were observed for analyses lasting > 48 weeks and for HBV. TAF was also associated with an improvement of SCr of 0.03 mg/dL (95% CI: 0.02 to 0.04) from treatment initiation compared to TDF (I^2^ = 82.47%) (Figure 2).

**Figure:**
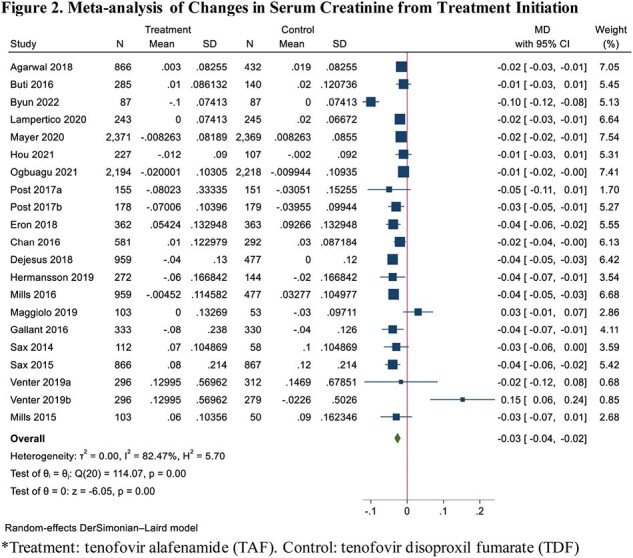

**Conclusion:**

This synthesis of RCT results supports a comparative renal safety advantage of TAF over TDF when evaluated across a broad and diverse range of people including those diagnosed with HIV-1, HBV, HIV and HBV coinfection, and those receiving PrEP. Further exploration of sources of heterogeneity and the evaluation of observational study data are needed.

**Disclosures:**

**Kyu Yun Park, PharmD**, Gilead Sciences, Inc.: Grant/Research Support **Connor Willis, PharmD**, ARUENA: Grant/Research Support|AstraZeneca: Grant/Research Support|Bayer: Grant/Research Support|Boehringer Ingelheim: Grant/Research Support|DexCom: Grant/Research Support|Gilead: Grant/Research Support|Gilead Sciences, Inc.: Grant/Research Support|Grail, Inc.: Grant/Research Support|Jazz: Grant/Research Support|Mainstay Medical: Grant/Research Support|Novartis: Grant/Research Support|PEAR Therapeutics: Grant/Research Support **Rachel Rogers**, Gilead Sciences, Inc.: Employee; Medical writing support provided by Aspire Scientific (Bollington, UK)|Gilead Sciences, Inc.: Stocks/Bonds (Private Company) **Amy Weinberg, DNP, MS**, Gilead Sciences, Inc.: Employee|Gilead Sciences, Inc.: Stocks/Bonds (Private Company) **Julia Green, MS, APRN, AGNP-C, ACRN, AAHIVE**, Gilead Sciences, Inc.: Employee|Gilead Sciences, Inc.: Stocks/Bonds (Private Company) **Aileen Chi, PharmD**, Gilead Sciences, Inc.: Employee|Gilead Sciences, Inc.: Stocks/Bonds (Private Company) **Nathorn Chaiyakunapruk, PhD**, Gilead Sciences, Inc.: Grant/Research Support

